# A Regulatory Roadmap for Repurposing: Comparing Pathways for Making Repurposed Drugs Available In The EU, UK, And US

**DOI:** 10.1017/jme.2024.171

**Published:** 2024

**Authors:** Mirre Scholte, Liam Bendicksen, Sabine E. Grimm, Teebah Abu-Zahra, Bianca Pauly, Manuela Joore, Aaron S. Kesselheim

**Affiliations:** 1: DEPARTMENT OF CLINICAL EPIDEMIOLOGY & MEDICAL TECHNOLOGY ASSESSMENT (KEMTA), MAASTRICHT UNIVERSITY MEDICAL CENTRE, MAASTRICHT, THE NETHERLANDS; 2: CARE AND PUBLIC HEALTH RESEARCH INSTITUTE (CAPHRI), MAASTRICHT UNIVERSITY, MAASTRICHT, THE NETHERLANDS; 3: DEPARTMENTS OF IQ HEALTH & RADIOLOGY, RADBOUDUMC, NIJMEGEN, THE NETHERLANDS; 4: PROGRAM ON REGULATION, THERAPEUTICS, AND LAW (PORTAL), DIVISION OF PHARMACOEPIDEMIOLOGY AND PHARMACOECONOMICS, DEPARTMENT OF MEDICINE, BRIGHAM & WOMEN’S HOSPITAL/HARVARD MEDICAL SCHOOL, BOSTON, MASSACHUSETTS, USA; 5: REGULATORY AFFAIRS DEPARTMENT, 3D-PHARMXCHANGE, TILBURG, THE NETHERLANDS

**Keywords:** Drug Repurposing, Prescription Drug Regulation, European Medicines Agency, US Food and Drug Administration, UK Medicines and Healthcare Products Regulatory Agency, Drug Approvals

## Abstract

To help academic and non-profit investigators interested in drug repurposing navigate regulatory approval processes, we compared pathways for repurposed drugs to obtain approval at EMA, UK MHRA, and the US FDA. Though we found no pathways specifically for repurposed drugs, pathways to market are available in all repurposing scenarios.

Drug repurposing entails discovering new therapeutic applications for drugs that were originally intended for other uses. Given that repurposed drugs have, in general, already undergone some degree of clinical testing, this practice can help expand the range of treatment options available to patients in a resource-efficient manner. Researchers can leverage existing knowledge of drugs’ safety profiles, effectiveness, pharmacokinetic activity, and other characteristics to move into a new clinical area.^1^ Prior research has shown that repurposed drugs made up over a third of transformative pharmaceuticals that came to market between 1984 and 2009.^2^

Despite some notable success stories, several factors may inhibit efforts to maximize the public health value of drug repurposing.^3^ One major issue is that drug repurposing may not align with for-profit companies’ bottom line. For instance, drug manufacturers may abandon promising compounds for market-driven considerations that are unrelated to safety or effectiveness.^4^ The profitability of repurposed drugs may be lower than that of new therapies, due to greater barriers to obtaining patents, the availability of lower-cost, substitutable generics, or other factors.^5^ Companies rarely pursue new indications for drugs that have been generic for a long time, even though this research concerns drugs with long-established safety profiles and can be cheaper than research on novel drugs.^6^

To obtain regulatory approval for a drug, a sponsor must demonstrate sufficient efficacy and safety to a drug regulator. That process usually involves conducting clinical trials that must meet certain basic standards, subjecting manufacturing facilities to oversight, and agreeing on a formal drug labeling. Few studies have examined the application of this standard regulatory process to repurposed drugs. Regulatory approval helps facilitate patient access to repurposed medicines, since payers’ coverage of on-label indications tends to be less restrictive than coverage of unapproved uses.

The EU has centralized (all EU member states and Iceland, Liechtenstein, and Norway, through EMA), decentralized or national, and mutual recognition procedures for drug approval. As of 2020, the UK has left the EU and the MHRA is now a standalone regulator. Drugs approved via reference regulators (amongst others the FDA or EMA centralized and national procedures) can obtain market access in the UK or Great Britain through the international recognition procedure by the MHRA.^7^ A UK- or Great Britain-specific market authorization can be granted via the MHRA, which generally mimics EU legislation at present.^8^

A structural barrier to repurposing is that drug approval processes in Europe and the US cater to organizations with the capital and institutional capacities of for-profit pharmaceutical companies, while drug repurposing is often pursued by academic institutions and not-for-profit entities.^9^ The process of obtaining approval requires substantial financial resources and regulatory expertise, which are characteristics that not-for-profit organizations may not possess. Under current laws and regulations, regulators in the US and Europe cannot unilaterally and independently add new efficacy indications to products’ labeling, which complicates repurposing efforts led by government agencies and not-for-profit entities, although the proposed EU drug legislation intends to address this (see **Appendix**).

Companies rarely pursue new indications for drugs that have been generic for a long time, even though this research concerns drugs with long-established safety profiles and can be cheaper than research on novel drugs.

To help drug repurposers — particularly those in not-for-profit and academic settings — navigate regulatory approval pathways, we provide an overview of available routes to market and associated evidence requirements within the US Food and Drug Administration (FDA), the Medicines and Healthcare products Regulatory Agency (MHRA) in the UK, and the centralized procedure of the European Medicines Agency (EMA).

## Identifying Pathways to Market and Types of Repurposed Drugs

We examined pathways to market in the US, UK, and in the EU via the centralized EMA procedure for three categories of repurposed drugs: investigational drugs not approved by regulators (shelved drugs), approved drugs currently covered by market exclusivities and/or active patents (new drugs), and drugs no longer protected by exclusivities or patents (off-patent drugs). We investigated each of these categories separately because prior research has shown that the presence of generic competition substantially impacts drug repurposing efforts^10^ and because the absence of any marketing authorization for a drug complicates repurposed drugs’ path to patients.

Using publicly available laws, regulations, and regulatory guidance, we extracted information about pathways to market for repurposed shelved drugs, new drugs, and off-patent drugs. From these documents, we extracted information concerning the availability of repurposing routes, potential benefits in terms of data or market exclusivities, and requirements for scientific research supporting approval. This study did not require approval from an institutional review board because it did not involve human subjects research.

## Pathways to Market for Repurposed Drugs

**Figure 1:**
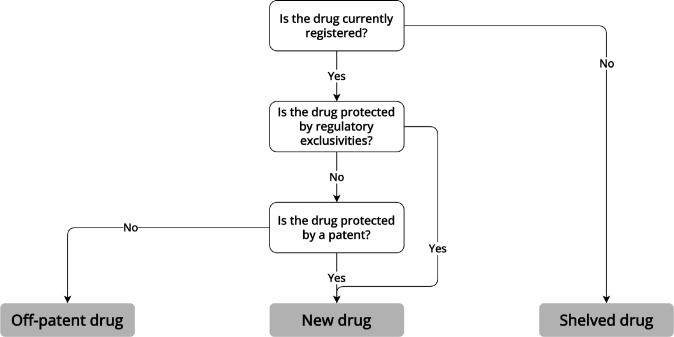
Drug repurposing scenarios Information extracted from laws, regulations, and regulatory guidance pertaining to the European Medicines Agency, UK Medicines and Healthcare products Regulatory Agency, and US Food and Drug Administration.

Pathways to approval for repurposed drugs vary based on the approval status of the drug and whether patents or exclusivities protect the product from competition (**Figure 1**). Depending on whether repurposing efforts concern shelved, new, or off-patent drugs, repurposers need to pursue different regulatory routes to come to market in the EU, UK, and the US. For each drug repurposing scenario, we discuss available regulatory pathways and evidence requirements and provide an illustrative example.

### Shelved Drugs

We define shelved drugs as drugs that were abandoned during research and development.^11^ Most have never received regulatory approval. Patent status varies: some shelved drugs are patented, for others patent protection has expired, and in some cases, developers never obtained patents.^12^

#### Considerations when Repurposing Shelved Drugs

**Figure 2:**
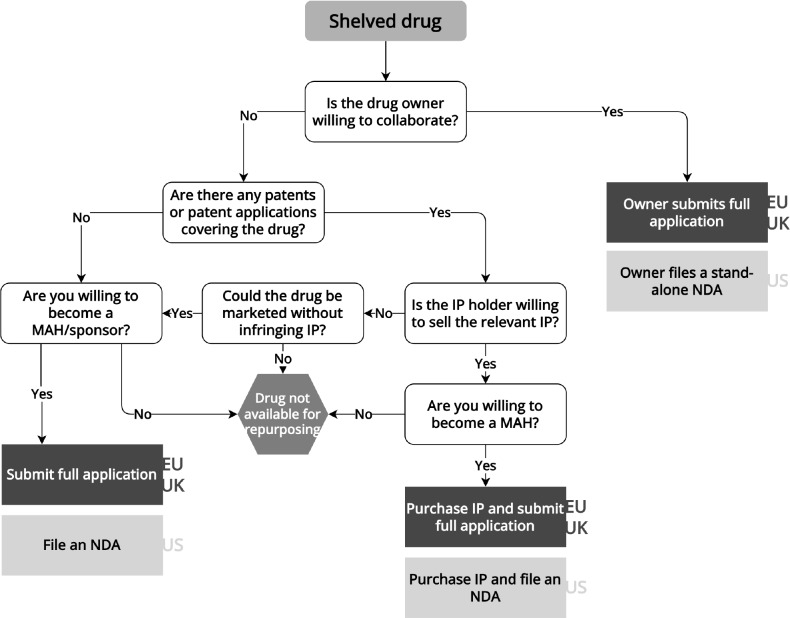
Flowchart for repurposing shelved drugs Information extracted from laws, regulations, and regulatory guidance pertaining to the European Medicines Agency, UK Medicines and Healthcare products Regulatory Agency, and US Food and Drug Administration. EU/UK regulatory pathways are shown in the dark gray boxes, while US pathways are shown in the light gray boxes. IP: intellectual property, MA: marketing authorization, MAH: marketing authorization holder, NDA: new drug application.

To repurpose a shelved drug, the repurposer (here the not-for-profit or academic initiator of the research) needs to liaise with the original drug developer, as it is likely that no other parties have access to the drug and data on these therapies are generally not publicly available. As a result, identifying shelved drugs for repurposing is a difficult undertaking.^13^ To seek regulatory approval for a shelved drug, repurposers may collaborate with the original developer, or the original developer may sell product-related rights and documentation to the repurposer (**Figure 2**). The latter option is often difficult because of liability concerns for the original developer, the time and costs required to acquire the necessary data, and the desire to keep products with therapeutic promise in-house.^14^ An example of a repurposed shelved drug is fexinidazole, which was originally discovered by Hoechst (now Sanofi) and later abandoned. The Drugs for Neglected Diseases initiative (DNDi) and collaborators repurposed the drug for sleeping sickness, a parasitic tropical disease. To develop, manufacture and distribute the drug, DNDi partnered with Sanofi.^15^ If the original drug developer is not willing to collaborate and the drug is not available elsewhere, shelved drugs generally cannot be repurposed in the EU, UK, and US.

#### Regulatory Pathways for Shelved Drugs

Previously shelved compounds seeking a repurposed indication can use regulatory pathways for new active substances within the EMA’s centralized procedure.^16^ When a marketing authorization is then granted, these drugs benefit from 8 years of data protection and 10 years of market exclusivity.^17^ UK market access can be granted on the basis of the “international recognition procedure” or via a “full application,” which mirrors EU regulations for new active substances and also has the same benefits.^18^ In the US, shelved therapies with active ingredients not previously approved by the FDA may receive approval via New Drug Applications (NDAs) for small-molecule drugs or Biologics License Applications (BLAs) for biologics. Under US law, small-molecule drugs containing new chemical entities receive 5 years of market exclusivity before an application for a generic can be submitted (which may then take another 1–2 years before it is approved);^19^ new biologics receive 12 years of market exclusivity.^20^ Market exclusivity or data protection periods may be extended in all three jurisdictions if indications concern pediatric populations, and periods may also be longer if the repurposed indication treats a rare disease.^21^

#### Evidence Requirements

Shelved drugs not previously considered for regulatory approval in a given jurisdiction must file novel applications for market access. New applications for approval need a full dossier (EU, UK) or NDA or BLA (US) demonstrating the drug’s manufacturing quality, safety, and efficacy in preclinical and clinical studies.^22^ Drugs intended to treat “serious” conditions may be eligible for expedited pathways to marketing authorization in all jurisdictions.^23^

In the US, drugs must generally demonstrate sufficient safety and substantial evidence of effectiveness in “adequate and well-controlled investigations.” Though FDA regulators previously preferred that sponsors complete two clinical trials establishing that a drug’s efficacy outweighed its safety concerns prior to full approval, the agency’s approval standards have loosened in recent decades such that over half of all drug approvals now occur based on a single pivotal trial.^24^ The accelerated approval pathway permits drugs for serious or life-threatening conditions to enter the market if sponsors show “an effect on a surrogate endpoint that is reasonably likely to predict clinical benefit.”^25^

Conditional approval pathways within EMA and MHRA allow for less comprehensive evidence at the time of initial authorization for drugs that fulfill an unmet medical need, provided that “the benefit of the drug’s immediate availability to patients is greater than the risk inherent in the fact that additional data are still required.”^26^

### New Drugs

New drugs are new active substances with only one marketing authorization holder (or sponsor in US) and are generally protected from competition by data exclusivities (EU, UK), market exclusivities (all jurisdictions), and/or patents (all jurisdictions).

#### Considerations for Repurposing New Drugs

**Figure 3:**
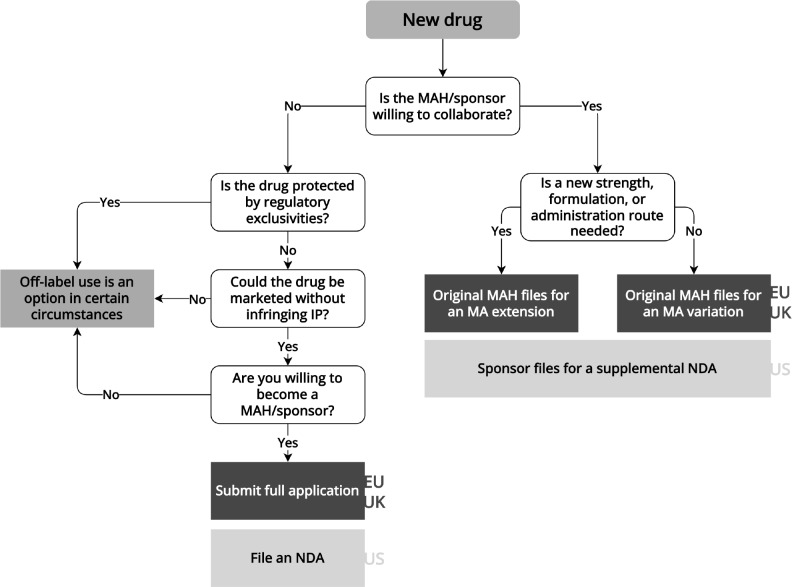
Flowchart for repurposing new drugs Information extracted from laws, regulations, and regulatory guidance pertaining to the European Medicines Agency, UK Medicines and Healthcare products Regulatory Agency, and US Food and Drug Administration. EU/UK regulatory pathways are shown in the dark gray boxes, while US pathways are shown in the light gray boxes. IP: intellectual property, MA: marketing authorization, MAH: marketing authorization holder, NDA: new drug application.

When repurposing new drugs, the repurposer needs to collaborate with the marketing authorization holder, which is generally also the intellectual property owner of the drug (**Figure 3**). For example, academic investigators successfully collaborated with marketing authorization holders to initiate the Drug Rediscovery Protocol (DRUP) study. In this study, 35 anticancer medicinal products, including those still protected by patents (e.g., pembrolizumab), were used off label to “treat treatment-exhausted patients with metastatic cancer that harbored an actionable oncogenic driver.”^27^

Sponsor-initiated new drug repurposing, namely adding supplemental indications to the labeling of a small molecule drug or biologic without generic or biosimilar competition, is common.^28^ Though such repurposing is valuable as a means of conducting formal regulatory reviews of the safety and efficacy of approved drugs in new indications, the addition of supplemental indications to a new drug’s label can be used to prolong manufacturers’ ability to charge high prices.^29^

#### Regulatory Pathways for New Drugs

Within the EMA and MHRA, marketing authorization holders can add indications to their market authorization via a variation or an extension application.^30^ If the drug is used in the same formulation, strength, and dosage as in the original marketing authorization, the market authorization holder can apply for a type II variation; otherwise, an extension is needed. If the repurposed indication is added to the market authorization within the first eight years after initial market authorization, and the new indication has “significant clinical benefit” relative to existing therapies, an additional year of market exclusivity may be granted.^31^ In the US, sponsors of small-molecule drugs and biologics may submit information about proposed new indications to the FDA in so-called efficacy supplements. These supplements, if approved, amend the sponsor’s existing BLA or NDA to add the novel indication. NDA sponsors may receive three years of market exclusivity for demonstrating efficacy in a repurposed indication.^32^ Per FDA regulations, efficacy supplements for drugs approved under an NDA that include an “investigation in humans the results of which have not been relied on by FDA” for other submissions to the agency may receive this three-year market exclusivity.

#### Evidence Requirements

In the EU and UK, the application for a repurposed new drug should demonstrate safety and efficacy of the drug in the new indication. In case of an extension, “the results of appropriate toxicological and pharmacological tests and/or appropriate clinical trials must be provided.” EMA and MHRA also evaluate if there is “significant clinical benefit” to determine whether one extra year of market exclusivity is appropriate. A new treatment is of significant clinical benefit if “it provides a clinically relevant advantage or major contribution to patient care in terms of greater efficacy, improved safety profile, and/or more favorable pharmacokinetic properties resulting in demonstrable clinical advantages compared to existing methods.”^33^

The FDA’s approval standard for new indications is the same bar as for a first approval: sponsors must demonstrate substantial evidence of efficacy and sufficient safety in adequate and well-controlled studies. However, prior research has shown that fewer pivotal trials of drugs’ efficacy supported supplemental indication approvals for small molecules and biologics compared to first approvals.^34^ Also, since sponsors have already demonstrated drugs’ safety profiles in other patient populations, fewer earlier-stage investigations such as pharmacokinetic and toxicity studies may be required.^35^ Repurposers’ primary challenge for obtaining FDA approval for new indications is demonstrating efficacy in a randomized, well-controlled trial.

### Off-Patent Drugs

Off-patent drugs are not covered by patents or market exclusivities.^36^ Multiple sponsors often manufacture off-patent generic drugs, though in some cases only the originator or one manufacturer markets an off-patent drug at a given time.

#### Considerations for Repurposing Off-Patent Drugs

**Figure 4:**
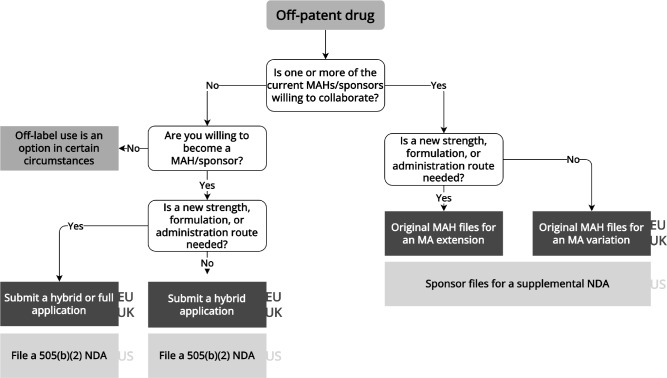
Flowchart for repurposing off-patent drugs Information extracted from laws, regulations, and regulatory guidance pertaining to the European Medicines Agency, UK Medicines and Healthcare products Regulatory Agency, and US Food and Drug Administration. EU/UK regulatory pathways are shown in the dark gray boxes, while US pathways are shown in the light grat boxes. MA: marketing authorization, MAH: marketing authorization holder, NDA: new drug application.

Since off-patent drugs hav e sometimes been on the market for decades, repurposers can leverage existing safety and effectiveness evidence to support new regulatory approvals (**Figure 4**). A recent example of off-patent repurposing is dexamethasone for the treatment of COVID-19.^37^ Dexamethasone is a glucocorticoid prescribed for a range of conditions.^38^ After a small, randomized trial indicated that another glucocorticoid, methylprednisolone, might benefit COVID patients,^39^ the RECOVERY group initiated a large-scale randomized trial. The RECOVERY trial’s success spurred the EMA to endorse dexamethasone’s utility for COVID patients on oxygen or mechanical ventilation.^40^ In 2020, a manufacturer applied for market authorization for dexamethasone, but this application was withdrawn in 2021 because the manufacturer was unable to remove preservatives from the medicine within the timeframe required by EMA. To date, regulators in the EU and US have not approved any dexamethasone product for use in patients with COVID.

#### Regulatory Pathways for Off-Patent Drugs

Generics generally must match the reference product’s label. Therefore, within the EU and UK, an existing originator, if willing to market the drug for the new indication, can broaden the scope of its market authorization via a variation or extension application.^41^ Typically no benefits in terms of market exclusivity or data protection apply. In case of extensive research into the new indication, however, one additional year of data protection may be granted.^42^ Alternatively, the repurposer can also consider becoming a marketing authorization holder by filing for a hybrid^43^ or full application^44^ within the EU or UK. In a hybrid application, parts of the application dossier (e.g., pre-clinical testing and safety) may be based on the reference medicinal product.^45^ In case of extensive research into the new indication and a hybrid application, one year of data protection may be granted.^46^ The EU has proposed a new pharmaceutical legislation, consisting of a regulation and a directive, which could potentially encourage drug repurposing, particularly for off-patent drugs (see **Appendix**).^47^

In the US, repurposed off-patent drugs can come to market via two distinct regulatory pathways. If the originator drug’s sponsor is willing to submit an efficacy supplement to the FDA that details the repurposed indication, it must sponsor, conduct, or rely upon “adequate and well-controlled investigations” that amount to “substantial evidence” of efficacy in the new indication.^48^ Alternatively, a new sponsor may submit a 505(b)(2) application to the FDA. This pathway allows repurposers to rely on safety data from the literature or data previously submitted by other manufacturers without the permission of those parties. The repurposer must still demonstrate “substantial evidence” of effectiveness in clinical trials, but does not need to have itself sponsored or conducted trials establishing efficacy in the new indication.^49^ The FDA may grant 505(b)(2) sponsors 3 years of market exclusivity if they have “conducted or sponsored the [pivotal] study by providing 50 percent of the funding or by purchasing exclusive rights to the study.”^50^

If the repurposer is not willing to market the drug, no current marketing authorization holder is willing to collaborate, and the drug is not available elsewhere, using the drug without formal regulatory approval may be the only option.

#### Evidence Requirements

In the EU and UK, the extent of additional studies required for hybrid applications for small-molecule drugs and biologics depends on the changes introduced and will be a matter of scientific assessment by the relevant competent authorities. To be eligible for one year of data protection, applicants for a new indication should provide evidence of “significant preclinical or clinical studies,” which means at least one confirmatory clinical trial versus a suitable comparator.^51^

To add a repurposed indication to an off-patent drug’s label in the US, sponsors must demonstrate efficacy in the new indication in a clinical trial. If an NDA or BLA holder is willing to add the indication to the drug’s label, the evidentiary standards used in new biologic or small molecule approvals apply. If a repurposer of a small-molecule drug chooses to file a 505(b)(2), the drug’s sponsor must conduct a pivotal trial showing that the product has efficacy in the new indication. In this instance, the bar to establish a product’s safety is easier to meet because repurposers can rely upon safety data submitted by sponsors that previously applied for regulatory approval for products containing the same active ingredient. Under the 21st Century Cures Act, sponsors of on-market biologics and small molecule drugs with established safety profiles may efficiently add supplemental indications to those drugs’ labels by submitting “qualified data summaries” from clinical trials to the FDA.^52^

## Discussion

Not-for-profit organizations and academic institutions interested in pursuing regulatory approval for repurposed drugs can use several pathways to market repurposed indications depending on whether the drug is shelved, off-patent, or protected by patents or exclusivities. These status-dependent pathways require different degrees of clinical testing and render repurposers eligible for different lengths of market exclusivity. Despite repurposing’s potential to increase patients’ treatment options in a resource-efficient manner, pharmaceutical regulations in the EU, UK, and US do not specifically and directly address drug repurposing, although the recently proposed reform of the EU pharmaceutical legislation will likely change this for some cases.

### Strengths and Limitations

Prior research has demonstrated a broad set of barriers in drug repurposing, including barriers in collaboration, research and development, and regulatory and financial barriers.^53^ This article describes regulatory routes and associated evidence requirements for repurposing in the EU, UK and US. Also, this article explicitly takes the viewpoint of not-for-profit and academic institutions and maps pathways from their perspectives, which we hope will help such organizations in choosing the most appropriate path.

A potential limitation of this study is that we only covered centralized regulatory routes for the EU, despite decentralized and national regulatory routes being available. This decision was made because the centralized procedure is mandatory for some drugs or indications and procedures may differ between countries. Second, we did not cover patenting concerns and strategies in depth in this study. The presence of a patent can complicate repurposing efforts.

### Potential Barriers and Opportunities

#### Reducing Unnecessary Off-Label Prescribing

Clinicians may prescribe a drug for a patient even if regulators have not approved it for the relevant indication. This so-called off-label prescribing may facilitate patient access, but foregoing approval procedures may interfere with payer reimbursement decisions and limit patient and provider knowledge of the costs and benefits of the repurposed indication.^54^ Accordingly, off-label prescribing should not be policymakers’ preferred method of facilitating patient access to repurposed drugs. We highlighted instances when off-label prescribing is the only option and recommend that these receive further attention.

#### Not-For-Profit Repurposing Barriers and Legislative Opportunities

Though pursuing regulatory approval for repurposed drugs can promote patient access and trigger reviews of products’ safety and effectiveness in new indications, repurposers may not be well equipped to pursue approval. Becoming a marketing authorization holder may not make sense for academic institutions as they often lack the capacity, expertise, or resources to gain regulatory approval or manufacture the drug.^55^ The proposed EU pharmaceutical legislation aims to support drug repurposing by allowing not-for-profit institutions to submit evidence that could bring repurposed indications on label and by offering extra data protection for repurposed indications (see **Appendix**). EMA is also experimenting with academic drug repurposing and the EU supports efforts to bridge the academic and regulatory worlds.^56^ In the US, Congress should allow the FDA to add supplemental indications in response to citizen petitions.^57^

#### The Need for Public Sector Investment in Drug Repurposing

In addition to efforts to streamline regulatory pathways for drug repurposing, policymakers should consider changes to facilitate the development and marketing of repurposed drugs. For example, government investment in repurposing generic drugs could help maximize the therapeutic utility of existing products. The lack of public sector funding for such repurposing is striking, given that generic drugs have well-established safety profiles, can generally be sourced from multiple producers, and are available at lower prices than brand-name drugs.

## Conclusion

Although no specific drug repurposing pathways exist in the US or Europe, regulatory routes are available in almost all cases but vary depending on whether the repurposed drug is shelved, off-patent, or protected by patents or exclusivities. In some cases, drugs cannot be repurposed because existing marketing authorization holders are not willing to provide access, collaborate in research, or manufacture and market products. Potential strategies to support drug repurposing entail creating workable collaborations between marketing authorization holders and repurposers, providing more governmental funding for drug repurposing research, and providing support for not-for-profit and academic repurposers.

## Figures and Tables

**Appendix Figure 1:**
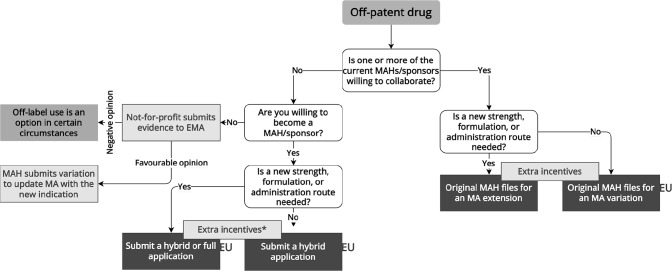
Flowchart for repurposing off-patent drugs under the proposed new EU pharmaceutical legislation EU/UK regulatory pathways are shown in the dark gray boxes, while US pathways are shown in the light gray boxes. *Extra incentives are only applicable to hybrid applications. EMA: European Medicines Agency, MA: marketing authorization, MAH: marketing authorization holder, NDA: new drug application.

## Previous Presentation

The authors presented a previous abstract of this article at the RExPO23 2023 conference in Stockholm, Sweden in October 2023. https://doi.org/10.58647/REXPO.23000027.v1

## Appendix Proposal for a New Pharmaceutical Legislation in the EU Related to Off-Patent Drug Repurposing

The EU has proposed a new pharmaceutical legislation, consisting of a regulation and a directive, which could facilitate the repurposing of off-patent drugs (**Appendix Figure 1**). ^1^ Via article 48 of the proposed regulation, not-for-profit entities may submit evidence concerning a new therapeutic indication of an authorized medicinal product that is expected to fulfill an unmet medical need. The European Medicines Agency will form an opinion on the risk-benefit ratio of the medicine and, in case of a favorable opinion, require the marketing authorization holders to update their marketing authorization with the new indication. Via article 84 of the proposed directive, medicinal products that have not previously benefited from data protection or 25 years have passed since the granting of the initial marketing authorization can be granted four additional years of data protection provided that significant clinical benefit is demonstrated in the new indication.
